# Correction: Steady states and kinetic modelling of the acid-catalysed ethanolysis of glucose, cellulose, and corn cob to ethyl levulinate

**DOI:** 10.1039/d4ya90045f

**Published:** 2024-11-08

**Authors:** Conall McNamara, Ailís O’Shea, Prajwal Rao, Andrew Ure, Leandro Ayarde-Henríquez, Mohammad Reza Ghaani, Andrew Ross, Stephen Dooley

**Affiliations:** a School of Physics, Trinity College Dublin Dublin 2 Ireland mcnamac4@tcd.ie; b School of Engineering, Department of Civil, Structural & Environmental Engineering Trinity College Dublin Dublin 2 Ireland; c School of Chemical and Process Engineering, University of Leeds 209 Clarendon Road Leeds LS2 9JT UK

## Abstract

Correction for ‘Steady states and kinetic modelling of the acid-catalysed ethanolysis of glucose, cellulose, and corn cob to ethyl levulinate’ by Conall McNamara *et al.*, *Energy Adv.*, 2024, **3**, 1439–1458, https://doi.org/10.1039/D4YA00043A.

The authors regret errors in the order of reaction conditions presented in Fig. 7 and 8. These errors do not impact the conclusions or interpretations provided in the text. In addition, the asterisks given in the original Fig. 7 and 8 communicate unnecessary additional information.

**Fig. 7:**
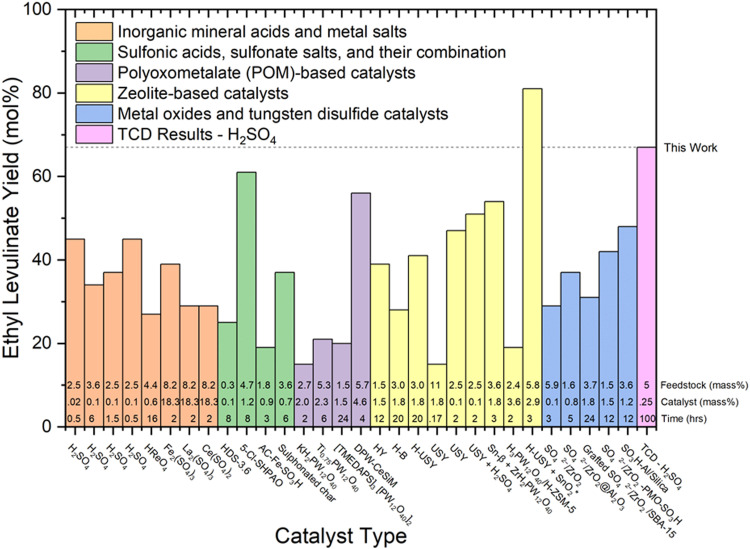
Literature review of experimental yields of ethyl levulinate using various catalyst types.^17,18,21,22,27,29,30,32,37–39,41–43,47,50,51,53,60,67–71^ All reaction systems use conventional heating, glucose as a feedstock, and a one-pot process. The feedstock loading (mass%), catalyst loading (mass%), and reaction times (hrs) are displayed at the bottom of each column.

**Fig. 8:**
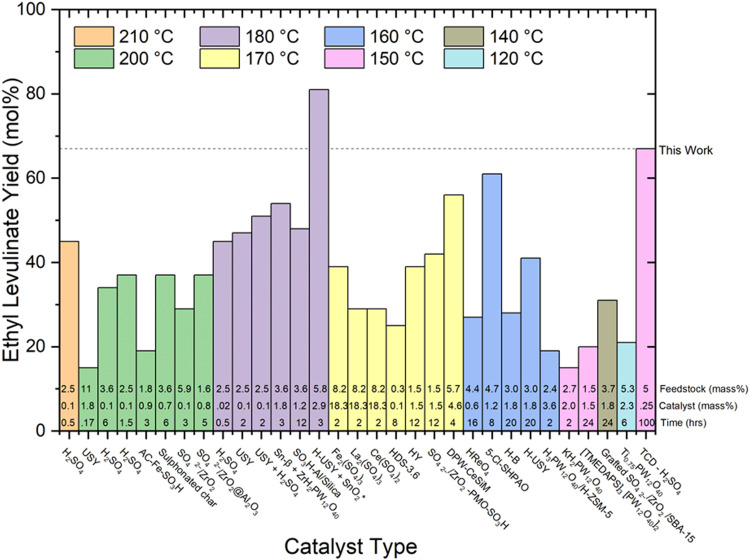
Literature review of experimental yields of ethyl levulinate using various catalyst types.^17,18,21,22,27,29,30,32,37–39,41–43,47,50,51,53,60,67–71^ All reaction systems use conventional heating, glucose as the feedstock, and a one-pot process. The feedstock loading (mass%), catalyst loading (mass%), and reaction times (hrs) are displayed at the bottom of each column.

The updated Fig. 7 and 8, along with their revised captions, are as follows.

The Royal Society of Chemistry apologises for these errors and any consequent inconvenience to authors and readers.

